# Complement Activation by C-Reactive Protein Is Critical for Protection of Mice Against Pneumococcal Infection

**DOI:** 10.3389/fimmu.2020.01812

**Published:** 2020-08-13

**Authors:** Sanjay K. Singh, Donald N. Ngwa, Alok Agrawal

**Affiliations:** Department of Biomedical Sciences, James H. Quillen College of Medicine, East Tennessee State University, Johnson City, TN, United States

**Keywords:** C-reactive protein, acute phase response, complement, inflammation, pneumococcal infection

## Abstract

C-reactive protein (CRP), a component of the innate immune system, is an antipneumococcal plasma protein. Human CRP has been shown to protect mice against infection with lethal doses of *Streptococcus pneumoniae* by decreasing bacteremia. *in vitro*, CRP binds to phosphocholine-containing substances, such as pneumococcal C-polysaccharide, in a Ca^2+^-dependent manner. Phosphocholine-complexed human CRP activates the complement system in both human and murine sera. The mechanism of antipneumococcal action of CRP *in vivo*, however, has not been defined yet. In this study, we tested a decades-old hypothesis that the complement-activating property of phosphocholine-complexed CRP contributes to protection of mice against pneumococcal infection. Our approach was to investigate a CRP mutant, incapable of activating murine complement, in mouse protection experiments. We employed site-directed mutagenesis of CRP, guided by its three-dimensional structure, and identified a mutant H38R which, unlike wild-type CRP, did not activate complement in murine serum. Substitution of His^38^ with Arg in CRP did not affect the pentameric structure of CRP, did not affect the binding of CRP to pneumococci, and did not decrease the stability of CRP in mouse circulation. Employing a murine model of pneumococcal infection, we found that passively administered H38R CRP failed to protect mice against infection. Infected mice injected with H38R CRP showed no reduction in bacteremia and did not survive longer, as opposed to infected mice treated with wild-type CRP. Thus, the hypothesis that complement activation by phosphocholine-complexed CRP is an antipneumococcal effector function was supported. We can conclude now that complement activation by phosphocholine-complexed CRP is indeed essential for CRP-mediated protection of mice against pneumococcal infection.

## Introduction

C-reactive protein (CRP) is a multifunctional component of the acute phase response and innate host defense machinery (1, 2). CRP is composed of five identical subunits arranged as a cyclic pentamer (3, 4). Each subunit has a phosphocholine (PCh)-binding site through which CRP binds to PCh-containing substances such as C-polysaccharide (PnC) of the cell wall of Streptococcus pneumoniae, in a Ca^2+^-dependent manner (3–6). After complexing with a ligand such as PnC, CRP activates the complement system (7, 8). Human CRP activates complement in both human and murine sera (9, 10). In human serum, CRP binds to C1q and activates the classical pathway of complement (7). Since human CRP does not interact with murine C1q, it is not known which pathway is utilized by human CRP to activate murine complement (9).

The C1q-binding site of CRP is formed in and around a cleft that is located on the opposite side of the PCh-binding site of the CRP pentamer (3, 4). The amino acid residues which contribute to the formation of the C1q-binding site of CRP are His^38^, Glu^88^, Asp^112^, Asn^158^, and Tyr^175^ from one subunit and Lys^114^ from the neighboring subunit. Mutational analysis of these amino acids revealed that His^38^, Asp^112^, and Tyr^175^ were critical for binding to C1q and activating complement in human serum (11, 12). Asp^112^ and Tyr^175^ appeared to be the C1q contact residues. Three CRP mutants, H38R, D112N, and Y175A have been previously identified as the mutants which displayed reduced binding to C1q and did not activate complement in human serum (12).

Human CRP has been shown to protect mice against lethal pneumococcal infection (13–16). Although a functioning complement system is required for full CRP-mediated protection, the exact mechanism of action of CRP in protecting mice against pneumococcal infection is not known (17–19). Decades ago, it was hypothesized that complement activation by CRP complexed with PCh was responsible for CRP-mediated protection of mice against pneumococcal infection (20). This hypothesis could not be tested experimentally at the time due to the unavailability of a CRP mutant which would bind to pneumococci but would not activate complement in murine serum.

Previously, we tested the Y175A CRP mutant for activation of murine complement. We reported that Y175A CRP did not activate human complement but activated murine complement (9). Other CRP mutants, H38R and D112N, that did not activate human complement were not tested for murine complement activation earlier. Here, we report that the CRP mutant H38R does not activate murine complement either. The availability of H38R CRP provided us with the needed tool to test the hypothesis that complement activation by PCh-complexed CRP is critical for CRP to protect mice against pneumococcal infection.

## Materials and Methods

### Construction and Expression of CRP Mutants

The construction of H38A and H38R CRP mutants has been described earlier (12). CRP mutants were expressed in CHO cells using the ExpiCHO Expression System (ThermoFisher Scientific) according to manufacturer's instructions. In brief, non-adherent ExpiCHO-S cells (Gibco) were cultured in a shaker flask at 37°C with 5% CO_2_. Cells (6 × 10^6^ cells/ml) were then transfected with mutant CRP cDNA (1 μg) using Expifectamine reagent (3.2 μl/ml). Transfected cells were cultured for 20 h at 37°C with 5% CO_2_. At 20 h post-transfection, ExpiCHO enhancer (6 μl/ml) and ExpiCHO feed (240 μl/ml) were added to the transfected cells and the culture was then transferred to 32°C with 8% CO_2_. The culture media containing expressed CRP mutants were harvested 14 days post-transfection.

### Purification of CRP

WT CRP was purified exactly as described previously, and the same method was used to purify CRP mutants H38A and H38R (21). In brief, CRP was purified by Ca^++^-dependent affinity chromatography on a PCh-Sepharose column (Pierce), followed by gel filtration on a Superose12 column (GE Healthcare) using the Biologic Duo Flow Protein Purification System (Bio-Rad). Purified CRP was stored in TBS (10 mM Tris-HCl, pH 7.2, containing 150 mM NaCl) containing 2 mM CaCl_2_ at 4°C and was used within 10 days. The purity and pentameric structure of CRP mutants were determined by SDS-PAGE and gel filtration.

For use in mice, purified CRP was treated with the Detoxi-Gel Endotoxin Removing Gel (ThermoFisher Scientific) according to manufacturer's instructions. The concentration of endotoxin in all CRP preparations, as determined by using the Limulus Amebocyte Lysate kit QCL-1000 (Lonza), was <2.2 endotoxin units per 25 μg CRP.

### Murine Complement Activation Assay

First, poly-L-lysine-PnC (P-PnC) was synthesized, as described previously (22), with some modifications. Briefly, 200 μl of 1 mg/ml PnC (Statens Serum Institute, 3459) was slowly added to 10 ml of 10 mM NaOH. Then, 10 mg of cyanuric chloride (Sigma, C95501) was added, followed by the addition of 2 ml of poly-L-lysine (200 μg/ml in H_2_O), to the mixture. After adjusting the pH to 8.2 using NaOH, the mixture was incubated for 2 h at 4°C with occasional stirring. The resulting P-PnC (poly-L-lysine ~20 μg/ml and PnC ~100 μg/ml) was stored at 4°C; a 1:4 dilution of this preparation was used to coat microtiter wells for the following assays.

Binding of CRP to the PCh ligand P-PnC was evaluated as follows: Microtiter wells were coated with P-PnC in 100 μl TBS, overnight at 4°C. The unreacted sites were blocked with TBS containing 0.5% gelatin for 1 h at room temperature. CRP, diluted in TBS containing 2 mM CaCl_2_, 0.1% gelatin and 0.02 % Tween 20 (TBS-Ca), was then added in duplicate wells and incubated for 2 h at 37°C. After washing the wells with TBS-Ca, bound CRP was detected by using anti-CRP monoclonal antibody HD2.4 diluted in TBS-Ca. HRP-conjugated goat anti-mouse IgG diluted in TBS-Ca was used as the secondary antibody. Color was developed using ABTS substrate and the OD was read at 405 nm in a plate reader.

Murine complement activation was assessed by measuring the deposition of activated murine C3 on P-PnC-complexed CRP, as follows: Microtiter wells were coated with P-PnC in 100 μl 10 mM phosphate buffer saline, pH 7.2 (PBS), overnight at 4°C. The unreacted sites were blocked with PBS containing 1% BSA for 1 h at room temperature, followed by rinsing the wells with buffer A (PBS containing 0.1% BSA and 1 mM CaC1_2_). CRP diluted in buffer B (buffer A containing 0.01% Tween 20) was then added in duplicate wells and incubated for 1 h at 37°C. The wells were washed with buffer B and then with buffer C (PBS containing 1% BSA, 0.15 mM CaC1_2_ and 0.5 mM MgC1_2_). Normal mouse serum (Innovative Research IGMSC57SER), diluted 1/30 in chilled buffer C, was added to each well and incubated for 30 min at 37°C, followed by washing with buffer C. Goat anti-mouse C3 antibody (Cappel), diluted 1/750 in buffer C, was added to each well. After 1 h at 37°C, the wells were washed, and developed with HRP-conjugated bovine anti-goat IgG (Santa Cruz Biotechnology). Color was developed using ABTS substrate and the OD was read at 405 nm in a plate reader.

### Pneumococcus Binding Assay

Pneumococcus binding assay was performed exactly as described previously (16, 23). Briefly, microtiter wells were coated with 10^7^ CFU of pneumococci overnight at 4°C. The unreacted sites in the wells were blocked with TBS containing 0.5% gelatin. CRP, diluted in TBS-Ca, was then added to the wells for 2 h at 37°C. After washing the wells with TBS-Ca, bound CRP was detected by using anti-CRP monoclonal antibody HD2.4. HRP-conjugated goat-anti mouse IgG was used as the secondary antibody. Color was developed using ABTS substrate and the OD was read at 405 nm in a plate reader.

### Clearance of H38R CRP From Mouse Circulation

The clearance rate of H38R CRP from the mouse blood was determined as described previously (23). Briefly, five mice were injected i.v. with 50 μg of H38R CRP in 100 μl TBS containing 2 mM CaCl_2_ through the tail vein. Blood samples were collected from the tip of the tail after 12, 16, 20, and 24 h, and sera were separated. The concentration of CRP in the sera was measured by ELISA.

### Mice

Male C57BL/6J mice, 8–10 weeks old, were purchased from Jackson Laboratories and used in the protection experiments. All animal studies have been reviewed and approved by the University Committee on Animal Care.

### Pneumococci

Virulent *S. pneumoniae* type 3, strain WU2 (obtained from Dr. David Briles, University of Alabama, Birmingham, AL), was cultured as described previously (23). Single use bacterial aliquot (1 ml) of virulent stock was prepared and stored at −80°C. For each experiment, an aliquot of frozen pneumococci was thawed in 50 ml Todd-Hewitt broth containing 0.5% yeast extract and incubated at 37°C with shaking at 125 rpm for 3 h and collected from mid-log phase cultures. The culture was centrifuged at 7,500 rpm for 15 min. The bacterial pellet was washed and resuspended in 10 ml normal saline and the volume adjusted to an absorbance A_600_ = 0.29 (3.5 × 10^8^ CFU/ml). The concentration, purity, and viability of pneumococci was confirmed by plating on sheep blood agar plates.

### Mouse Protection Experiments

Mouse protection experiments were performed exactly as described previously (24). In brief, mice were injected i.v. with 25 μg CRP. After 30 min, 100 μl of 3.4 × 10^8^ CFU/ml of pneumococci was injected. Survival of mice was recorded three times per day for 7 days. To determine bacteremia (CFU/ml), blood samples were collected from each surviving mouse twice daily for the first 3 days, followed by once daily for next 2 days. Blood was diluted and plated on blood agar plates and incubated for 18 h at 37°C before the colonies were counted. The plotting and statistical analyses of the data were done using the GraphPad Prism 4 software. Statistical significance for survival among the groups was determined by Log-rank test and differences in bacteremia were analyzed by Mann-Whitney test.

## Results

All experiments were performed three times, unless otherwise mentioned, and comparable results were obtained each time. Results of a representative experiment are shown in the figures where the raw data (OD_280_ or A_405_) were used to plot the curves.

### H38R CRP Does Not Activate Murine Complement

**Figure 1:**
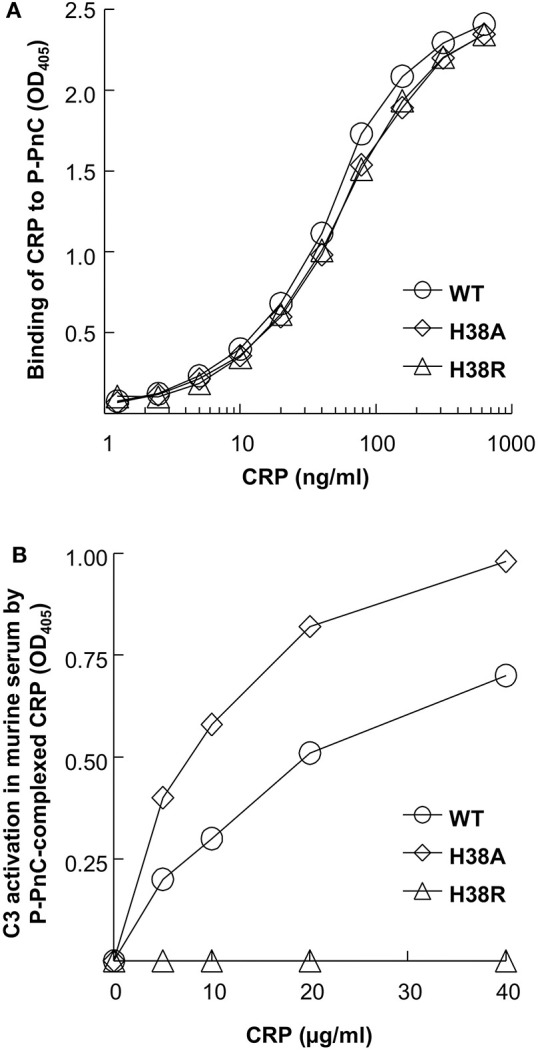
Activation of murine complement by human CRP. A representative of three experiments is shown. **(A)** Binding of CRP to P-PnC. Microtiter wells were coated with P-PnC. CRP diluted in TBS-Ca was added to the wells. Bound CRP was detected by using an anti-CRP antibody. Color was developed and the OD was read at 405 nm. **(B)** Activation of murine C3 by CRP complexed with P-PnC. Microtiter wells were coated with P-PnC. CRP diluted in TBS-Ca was added to the wells. Normal mouse serum was then added to the wells. Deposited C3 was detected by using goat anti-mouse C3 antibody. Color was developed and the OD was read at 405 nm.

Previously, for murine C3 deposition assays, we used CRP-PnC complexes to activate complement (9). However, we failed to generate a reliable murine C3 deposition assay using commercially available batch of PnC at this time. Instead of using CRP-PnC complexes, we used CRP-P-PnC complexes for murine C3 activation. As shown (Figure 1A), H38A and H38R CRP mutants bound to P-PnC as well as WT CRP did. In the C3 deposition assay (Figure 1B), WT CRP activated murine C3 in a CRP concentration-dependent manner. Like WT CRP, H38A CRP also activated murine C3 in a CRP concentration-dependent manner. Even if the binding of WT CRP and H38A CRP to P-PnC did not differ from each other, H38A CRP was more efficient than WT CRP in activating murine C3. However, H38R CRP did not result in any C3 deposition on CRP-P-PnC, suggesting that H38R CRP was unable to activate murine complement.

### H38R CRP Is Pentameric and Binds to Pneumococci

**Figure 2:**
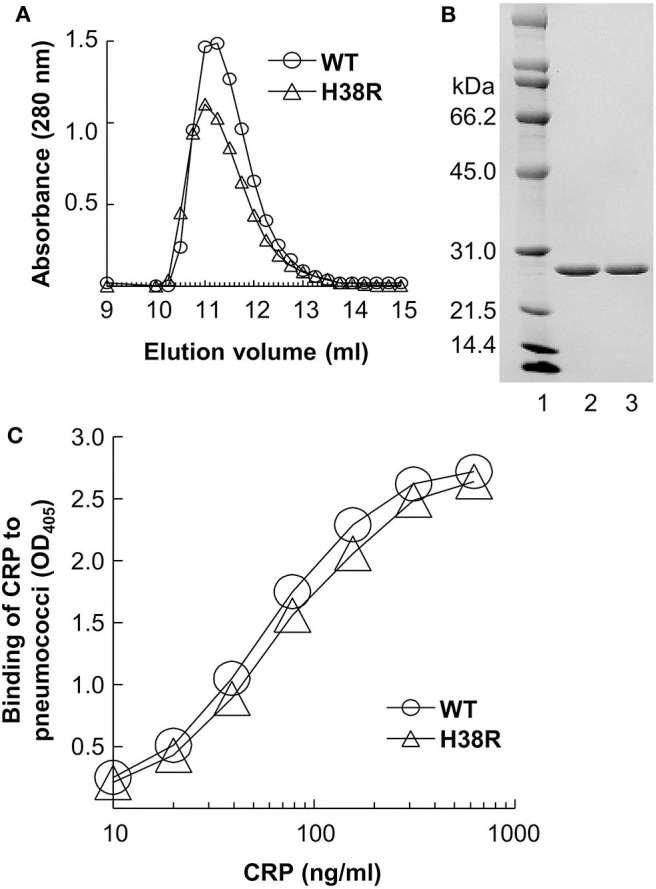
Overall pentameric structure of H38R CRP. A representative of three experiments is shown. **(A)** Elution profiles of CRP from the gel filtration column are shown. CRP in TBS containing 2 mM CaCl_2_ was applied to the column and eluted with the same buffer. Sixty fractions (0.25 ml) were collected and protein measured (A_280_) to determine the elution volume of CRP. **(B)** Denaturing SDS-PAGE of CRP. A Coomassie brilliant blue-stained gel (lane 2, WT CRP; lane 3, H38R CRP), is shown. **(C)** Binding of CRP to pneumococci. Microtiter wells were coated with pneumococci. CRP diluted in TBS-Ca was added to the wells. Bound CRP was detected by using an anti-CRP antibody. Color was developed and the OD was read at 405 nm.

The elution volume of H38R CRP from the gel filtration column was identical to that of WT CRP (Figure 2A), indicating that the molecular weight of H38R CRP was same as WT CRP. SDS-PAGE analysis (Figure 2B) of H38R CRP confirmed the purity of the preparation and showed that there was no difference in the molecular weight of the subunits of WT and H38R CRP. Thus, H38R CRP was pentameric. Also, the Ca^2+^-dependent binding of H38R CRP to pneumococci was similar to that of WT CRP (Figure 2C). We have reported previously that the Ca^2+^-dependent binding of H38R CRP to PnC and PCh-conjugated BSA was also similar to that of WT CRP (12).

### H38R CRP Is Stable *in vivo*

**Figure 3:**
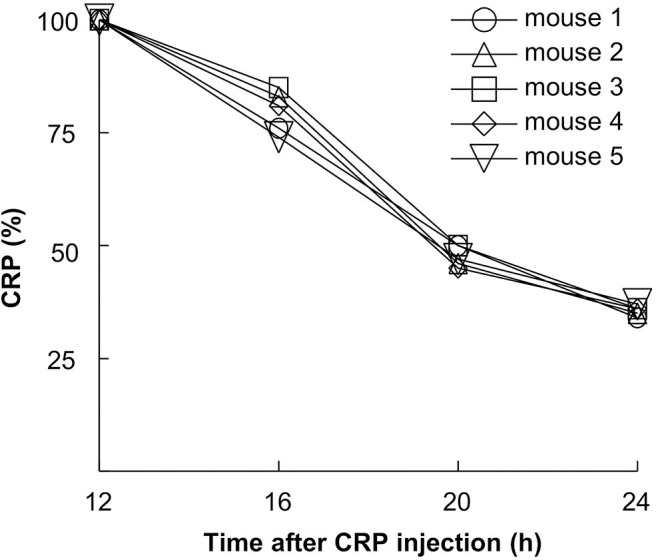
Clearance of H38R CRP from mouse circulation. Mice were injected with 50 μg of CRP. Blood was collected at various time points, sera separated, and the concentration of CRP measured.

We have reported previously that the rate of clearance of WT CRP from mouse circulation was 0.67 μg/ml/h (23). To determine the dose of H38R CRP for *in vivo* use, we evaluated the rate of clearance of H38R CRP from mouse circulation (Figure 3). The clearance rate of H38R CRP was found to be 0.20 μg/ml/h, suggesting that the clearance of H38R CRP was not faster than that of WT CRP and that the substitution of His^38^ with Arg did not reduce the stability of H38R CRP *in vivo*.

### H38R CRP Does Not Protect Mice Against Pneumococcal Infection

**Figure 4:**
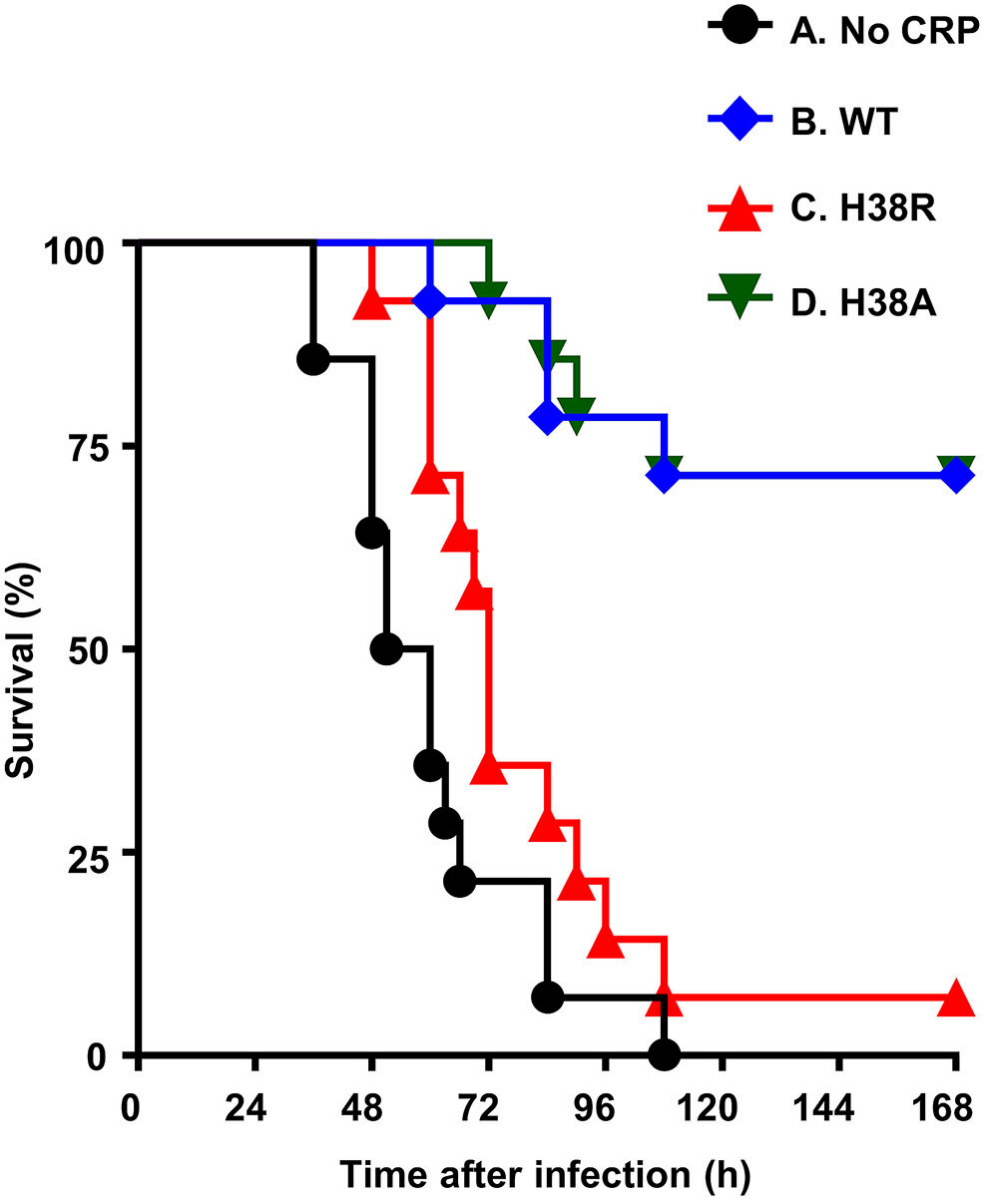
Survival curves of mice infected with pneumococci with and without CRP. CRP was injected first; pneumococci were injected 30 min later. The data are combined from two separate experiments with seven mice in each group in each experiment. The *p*-values for the differences in the survival curves between groups A B, A D, B C, and C D were <0.05. The *p*-values for the differences in the survival curves between groups A C and B D were >0.05.

Figure 4 shows the combined results from two separate mouse protection experiments. H38A CRP, which was not different from WT CRP in activating murine complement, was included as a control in the experiment. The median survival time (MST, the time taken for the death of 50% of mice) for mice injected with bacteria alone was 56 h. The MST for mice injected with H38R CRP was 72 h. There was no statistically significant difference between mice receiving H38R CRP and mice not receiving any CRP. The MST for mice injected with either WT CRP or H38A CRP could not be determined since >50% mice survived in both groups. There was no statistically significant difference between mice receiving either WT or H38A CRP.

**Figure 5:**
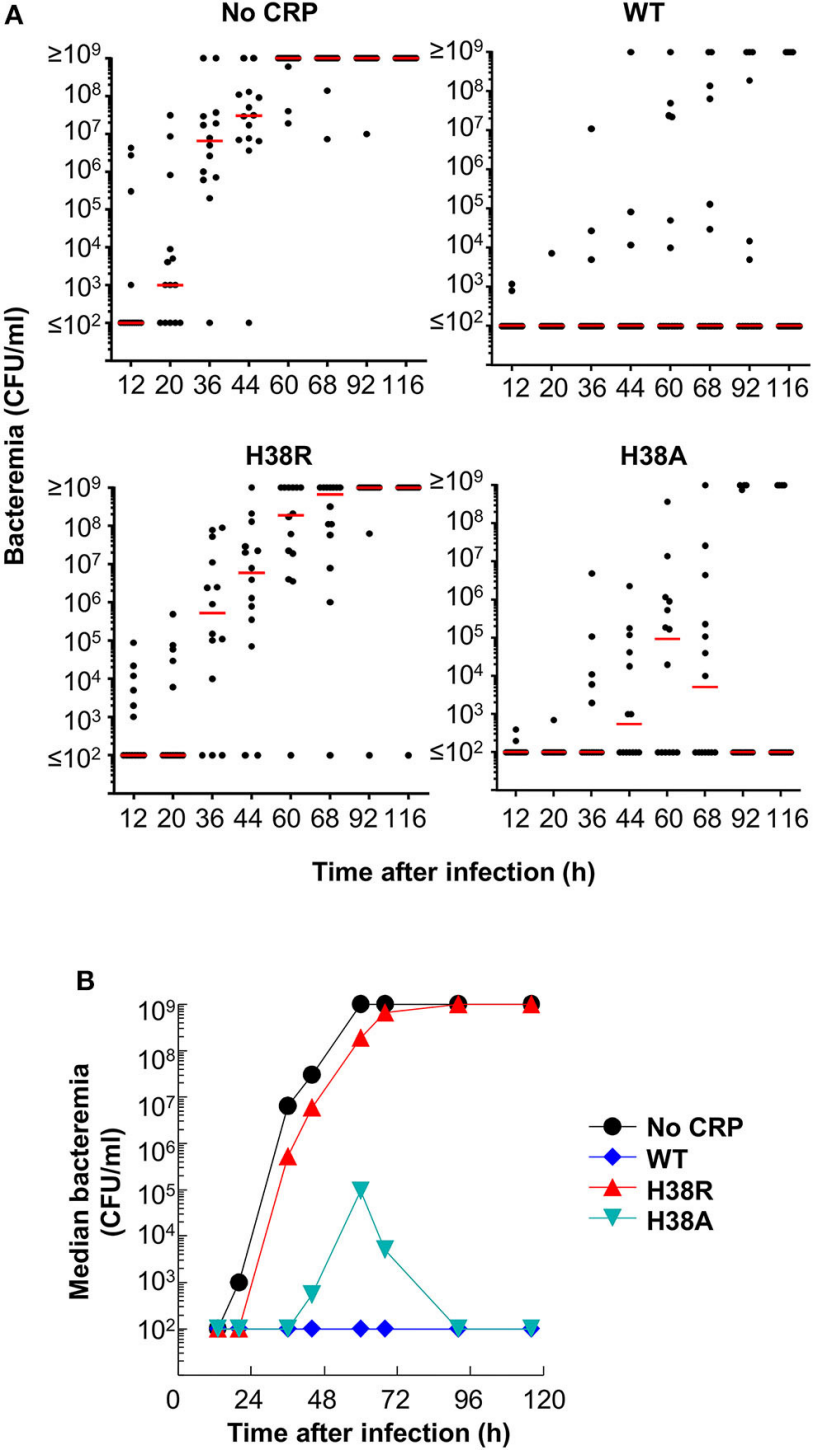
Bacteremia in mice infected with pneumococci with and without CRP. **(A)** Blood was collected from each surviving mouse shown in Figure 4. Bacteremia was determined by plating. Each dot represents one mouse. The horizontal line in each group of mice represents median bacteremia. A bacteremia value of >10^8^ indicates a dead mouse. The *p*-values for the differences between groups A B and A D were <0.05. The *p* value for the difference between groups A C was >0.05. **(B)** The median bacteremia values for each group shown in **(A)** are plotted.

Next, we determined bacteremia in each surviving mouse (Figure 5). In mice receiving H38A CRP, bacteremia decreased, like in WT CRP-treated mice. There was no statistically significant difference in bacteremia in WT CRP-treated and H38A CRP-treated mice. However, bacteremia continued to increase in H38R CRP-treated mice, like in untreated mice, and mice died once bacteremia was >10^8^ CFU/ml. There was no statistically significant difference in bacteremia in untreated and H38R CRP-treated mice. Combined data from survival of mice and bacteremia suggested that H38R CRP was not protective against pneumococcal infection and that the lethality of H38R CRP-treated mice was due to the inability of H38R CRP to decrease bacteremia.

## Discussion

Our major findings in this study were: 1. H38R CRP, which did not activate complement in human serum as reported previously (12), did not activate complement in murine serum either. 2. H38R CRP incapable of activating murine complement failed to protect mice against lethal pneumococcal infection. The inability of H38R CRP to protect mice against pneumococcal infection was solely due to its inability to activate the complement system since the H38R mutation did not reduce the stability of CRP; H38R CRP was more stable than WT CRP *in vivo*. Our findings confirm that complement activation by CRP-PCh complexes constitute the mechanism of CRP-mediated protection (decrease in bacteremia and increase in survival time) of mice against lethal pneumococcal infection.

Previously, we investigated the role of the PCh-binding site of CRP in protection of mice against pneumococcal infection employing a CRP triple mutant, F66A/T76Y/E81A, incapable of binding to PCh and pneumococci. Different mouse models provided different results (23, 24). Surprisingly, in one mouse model, CRP triple mutant protected mice against infection despite being unable to bind to PCh, suggesting that complement activation by CRP-PCh complexes was not required for protection (24). Later, we found out that CRP triple mutant, like acidic pH-treated WT CRP, had inadvertently gained the capability to bind to any protein that was immobilized on a polystyrene surface, including complement factor H (unpublished observations) (25, 26). Factor H is an inhibitor of complement activation and pneumococci recruit factor H to escape complement-dependent killing (27). Our current finding that complement activation by CRP-PCh complexes is absolutely required for protection suggests that in the previously published protection experiments involving CRP triple mutant (incapable of binding to PCh but capable of binding to immobilized factor H), at some point during the decrease in bacteremia, endogenous murine CRP might have participated in protection by binding to PCh on pneumococci and activating the murine complement system (24).

Human CRP activates complement in both human and murine sera (9, 10). Human CRP binds to C1q and activates the classical pathway of complement in human serum (7). It is not known whether the classical pathway is the only pathway through which human CRP can activate human complement. Human CRP does not interact with murine C1q and, therefore, the activation of murine complement by human CRP is not through the classical pathway (9). The pathway through which human CRP activates murine C3 remains undefined (9, 10). Based on the known crosstalk among CRP, lectins, ficolins, and pneumococci, it was proposed earlier that human CRP can activate complement through the lectin pathway also (9, 10, 28, 29). Irrespective of the pathway through which human CRP activates murine complement, our data suggest that the cleft on CRP, that accommodates the binding site for human C1q, is critical for human CRP to activate murine complement. However, all three amino acid residues, His^38^, Asp^112^, and Tyr^175^, critical for the formation of the binding site for human C1q and for activation of human complement, are not critical for murine complement activation. The Y175A CRP does not activate human complement but activates murine complement. The H38R does not activate complement in both human and murine sera. The D112N CRP does not activate human complement and has not been tested for murine complement activation yet. The role of the other amino acid residues, Glu^88^ and Asn^158^, present in the CRP cleft in activating murine complement is also unknown.

Despite several unanswered questions regarding the mechanisms of complement activation by human CRP in human and murine sera, we conclude that CRP cannot protect against pneumococcal infection if CRP is unable to activate the complement system. Also, since endogenous murine CRP has been shown to be protective against pneumococcal infection in another mouse model (2), we propose that the experiments on structure-function relationships of CRP in pneumococcal infection employing human CRP mutants should always be conducted employing CRP knockout mice (30).

## Data Availability Statement

All datasets presented in this study are included in the article.

## Ethics Statement

All animal studies have been reviewed and approved by the East Tennessee State University Committee on Animal Care.

## Author Contributions

SS and DN performed the experiments. AA conceived and designed the experiments. SS, DN, and AA analyzed the data and drafted the manuscript. All authors contributed to the article and approved the submitted version.

## Conflict of Interest

The authors declare that the research was conducted in the absence of any commercial or financial relationships that could be construed as a potential conflict of interest.

## Abbreviations

CRP: C-reactive protein
PCh: phosphocholine
PnC: pneumococcal C-polysaccharide
P-PnC: poly-L-lysine-conjugated PnC
WT: wild-type.
